# Quantitative optical coherence tomography angiography assessments in the fellow eyes of those with central serous chorioretinopathy

**DOI:** 10.1186/s12886-024-03549-9

**Published:** 2024-07-18

**Authors:** Jingjing Lin, Jianbo Mao, Shixin Zhao, Yiqi Chen, Lijun Shen

**Affiliations:** 1 Zhejiang Provincial People’s Hospital, 158 Shangtang Road Zhejiang Province, Hangzhou City, 310000 China; 2 National Clinical Research Center of Ocular Diseases, Eye Hospital, Wenzhou Medical University, Hangzhou, 310000 China; 3 Department of Optometry and Vision Sciences, The University of Melbourne, Parkville, VIC 3010 Australia

**Keywords:** Central serous chorioretinopathy (CSC), Optical coherence tomography (OCT), OCT angiography (OCTA), Retinal capillary vessel density (VD), Foveal avascular zone (FAZ)

## Abstract

**Background:**

To analyze the vessel density (VD) of the retina and choriocapillaris (CC) layer and the structure of the foveal avascular zone (FAZ) in the fellow eyes of central serous chorioretinopathy (CSC) patients by using optical coherence tomography angiography (OCTA).

**Methods:**

This was a case–control study. Unilateral CSC patients and age-matched healthy subjects were recruited from the Affiliated Eye Hospital of Wenzhou Medical University between July 2016 and July 2021. All eyes were divided into three groups: acute CSC (aCSC), chronic CSC (cCSC), and healthy controls. Both aCSC and cCSC were again divided into two subgroups: the affected eyes and the fellow eyes. In this study, all parameters of VD and FAZ were measured by self-software of OCTA.

**Results:**

A total of 231 eyes of 137 subjects were included, with 47 aCSC patients, 47 cCSC patients, and 43 healthy controls. In the fellow eyes of CSC, the retinal VD was significantly lower (all *P* < 0.05), and the FAZ was significantly larger (all *P* < 0.05) in the cCSC group than in healthy controls, while no difference was detected in the CC layer. There was no significant difference between the aCSC group and healthy controls in all OCTA parameters. In the affected eyes of CSC, the superficial retinal vessel density (SRVD) was significantly higher (all *P* < 0.05) in healthy controls than in the aCSC and cCSC groups, while the deep retinal vessel density (DRVD) was significantly lower (all *P* < 0.05) and the FAZ was larger (all *P* < 0.05) in the cCSC group than in the aCSC group and healthy controls. A liner regression equation was established: Y (BCVA, best corrected visual acuity) = 3.692–0.036✱X1 (DRVD-Fovea)-0.031✱X2 (FD-300, vessel density around the 300 μm width of the FAZ), R^2^ = 0.427.

**Conclusion:**

Based on OCTA measurements, this study revealed that the retinal microvascular network was impaired even in the fellow eyes of those with cCSC, which should arouse attention to the observation of unilateral CSC.

## Background

Central serous chorioretinopathy (CSC) is characterized by focal retinal pigment epithelium (RPE) or neurosensory detachment in the macular area, with mild to moderate visual impairment [1]. Venous overload choroidopathy and increased scleral thickness, RPE dysfunction, and upregulation of corticosteroids are three possible pathological mechanisms underlying CSC disease [1]. Recent research implies that CSC is more likely to be bilateral, whether in symptom or fundus structure. In a clinical study of 811 patients, up to 42.1% of CSC cases were bilateral, and the rate of bilateralism increased with longer follow-up [2]. Though the fellow eyes in CSC patients were asymptomatic and exhibited intact retinal structure, they showed considerable incidence of fluorescein leaking (31.42%) in a fundus fluorescein angiogram (FFA) [3]. Furthermore, significantly higher choroidal thickness [4] and choroidal vessel diameter were detected [5], while lower choriocapillaris (CC) blood flow [6]was observed in the fellow eyes. Moreover, approximately 25% of the fellow eyes with secondary choroidal neovascularization (CNV) showed subclinical vascular networks [7].

Recently, the advent of optical coherence tomography angiography (OCT-Angio, OCTA) enables to capture of the vessel density (VD) of the retina (both in the superficial and deep capillary plexus) and the CC layer, and the foveal avascular zone (FAZ) morphologically and quantitatively [6, 8, 9]. By using OCTA, our former study showed both more decreased VD and more expanded FAZ in the affected eyes of chronic CSC (cCSC) than acute CSC (aCSC) patients and healthy people, which were related to a lower best corrected visual acuity (BCVA) [10]. We now wonder whether these asymptomatic eyes of CSC patients in different stages may have been subclinical, in which case, misunderstanding the condition would influence the follow-up observation and even delay prompt therapy. Current studies involving the comprehensive parameters of fundus microstructure in both affected and fellow eyes of CSC patients have been few.

In this study, we performed OCTA both on the affected and fellow eyes of CSC patients and age-controlled healthy subjects, to investigate VD parameters of the retina and the CC layer, and FAZ parameters among the aCSC group, the cCSC group, and healthy controls and to determine their correlation with VA.

## Methods

This retrospective case–control study was approved by the Institutional Review Board and Ethics Committee of the Eye Hospital of Wenzhou Medical University. All participants, including CSC patients, healthy controls, and researchers, adhered to the tenets of the Declaration of Helsinki (No.121-K-107–01). Written informed consent was obtained from patients and controlled subjects after an explanation of the nature and possible consequences of the study.

All study subjects were recruited from the Affiliated Eye Hospital of Wenzhou Medical University between July 2016 and July 2021. Those with unilateral CSC (with only one eye affected) at the initial examination were included in the study. CSC was diagnosed as one or more serous retinal detachments (SRDs) or pigmented epithelium detachments (PEDs) in the macular area as detected by OCT, fluorescein leakage during FFA, and abnormal dilated choroidal vasculature detected by indocyanine green angiography (ICGA). Age-controlled patients with no previous systemic diseases or ocular diseases were enrolled.

The exclusion criteria were as follows: (1) a previous or existing history of glucocorticoid use or a state of pregnancy; (2) previous visit at other hospital or previous ocular surgery history; (3) other eye diseases that may affect retinal microvascular flow, such as moderate to severe cataract, diabetic retinopathy, age-related macular degeneration, glaucoma, or a macular epiretinal membrane; (4) previous treatments for CSC, including photodynamic therapy (PDT), photocoagulation, or intravitreal injections of anti-vascular endothelial growth factor (anti-VEGF) drugs; (5) high diopter > -6D or long eye axis > 26.0 mm; and (8) OCTA image quality below 7/10.

All study subjects underwent a complete ocular examination on the same day of their first visit, including a slit-lamp examination, an intraocular pressure test, BCVA, fundus photography, structural OCT, FFA, ICGA (Spectralis HRA + OCT; Heidelberg Engineering, Heidelberg, Germany), and OCTA (RTVue XR Avanti; Optovue Inc., Fremont, CA, USA). All eyes of the study subjects were divided into three groups: aCSC, cCSC, and healthy controls. The aCSC group was defined when patients had an onset of symptoms or SRD within 6 months, while the cCSC group had SRD, RPE changes, or active FA leakage for more than 6 months [1]. Age-controlled patients were enrolled with no previous systemic diseases or ocular diseases, and all the right eyes were collected. Both aCSC and cCSC groups were further divided into two subgroups: the affected eyes and the fellow eyes.

**Fig. 1:**
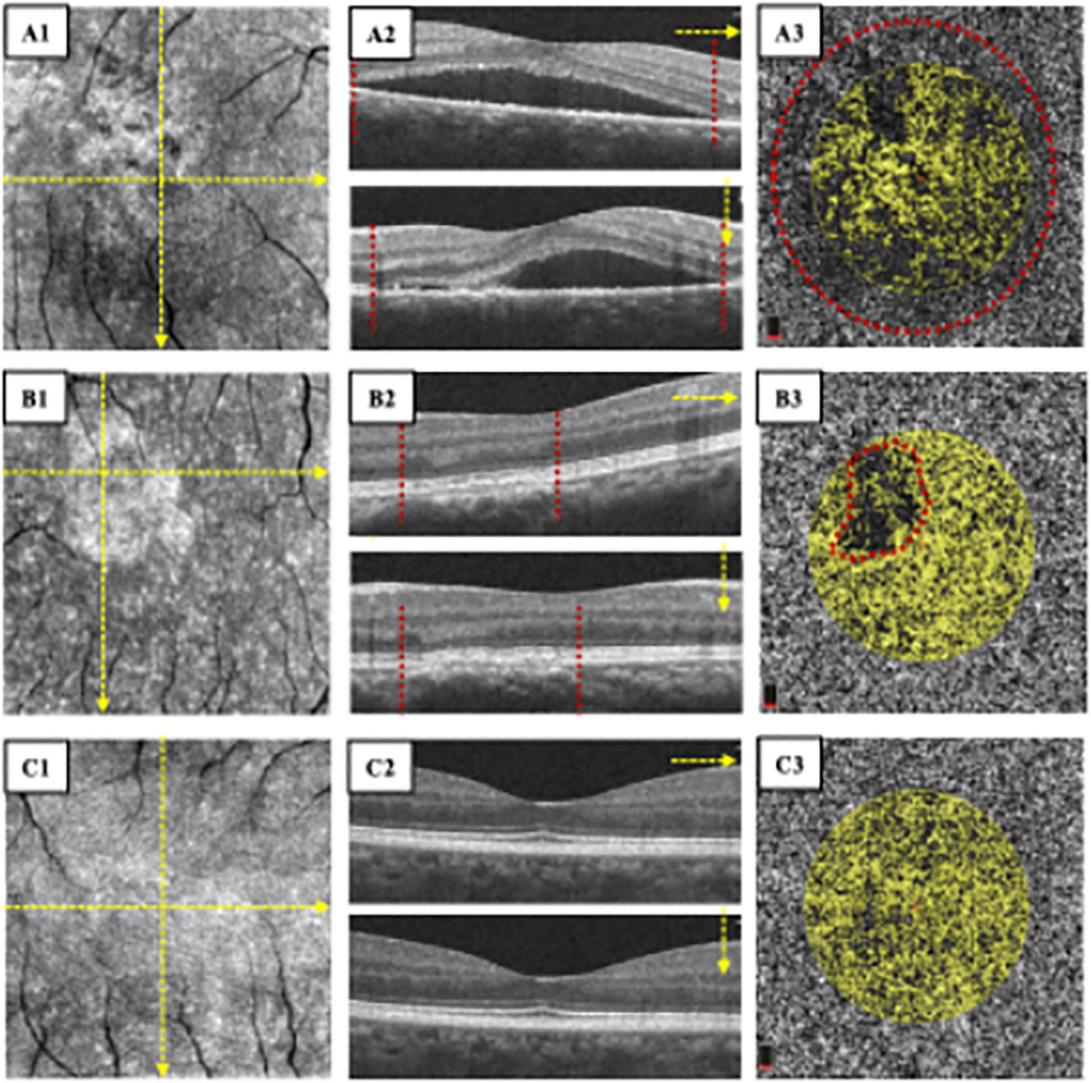
OCT and OCTA images of aCSC, cCSC, and healthy controls. A1-A4: healthy controls; B1-B4, C1-C4: the affected eye and the unaffected fellow eye of acute CSC patient, respectively; D1-D4, E1-E4: the affected eye and the unaffected fellow eye of chronic CSC patient, respectively

OCTA (3 mm X 3 mm) was performed on all study subjects. By using OCTA self-software (version 2017.1.0.155), the enface OCT image was automatically segmented into four layers [11]: superficial (from the inner surface of the internal limiting membrane to the outer surface of the inner plexiform layer—10 µm), deep (from the outer surface of the inner plexiform layer—10 µm to the inner surface of the outer plexiform layer + 10 µm), outer retina, and CC level (between two Bruch’s membrane segmentation lines with -9 and -31 µm offset). When different layer boundaries were obscure in the presence of large or irregular SRDs, we artificially adjusted and redefined the lines. The images with the unalterable false layer were excluded. In this study, OCTA variables, including the VD of the superficial and deep retina, the FAZ Area, the perimeter of FAZ (PERIM), the A-circularity index (AI, perimeter/standard circle perimeter with equal area), the FD-300 (vessel density around the 300 μm width of the FAZ), and the total area of flow signal within 1.00 mm radius from the center of the CC layer were collected by the built-in software. VD was defined as the ratio of the area of vessels divided by the total area measured in particular regions. The whole, fovea, and parafovea VD on the superficial retina (SRVD-whole, SRVD-fovea, and SRVD-parafovea) on the deep retina (DRVD-whole, DRVD-fovea, and DRVD-parafovea) were VD within 3 mm X 3 mm, 1 mm X 1 mm, and an annulus between 3 mm X 3 mm and 1 mm X 1 mm, respectively. To assess the VD of the CC layer, we first excluded images whose SRD areas were more than 1.00 mm radius from the center of the CC layer (Fig. 1 A1-A3) based on the enface image and OCT scan. Secondly, after excluding regions of SRD or irregular RPE alterations (Fig. 1 B3, red circle), we calculated the percentage of the remaining flow area to the remaining CC layer area as VD.

Each OCT image overlays 100 times, with an acquisition of a 30° area of the fovea. Central foveal thickness (CFT) and subfoveal choroidal thickness (SFCT) were acquired from the OCT image and collected twice from horizontal and vertical directions to obtain the mean value. The CFT was defined as the distance from the inner surface of the inner limiting membrane to the inner surface of the Bruch membrane at the fovea center. The SFCT was defined as the distance from the inner surface of the Bruch membrane to the innermost choroid-sclera interface. The intact external limiting membrane (ELM) or ellipsoid zone (EZ) and the regular RPE were recorded as ELM (-), EZ (-), and RPE (-), respectively, while the rupture of ELM or EZ, and irregular RPE alteration were recorded as ELM ( +), EZ ( +), and RPE ( +).

## Statistical analysis

All statistical analyses were conducted using SPSS software (version 26.0; SPSS, Chicago, IL, USA) and GraphPad Prism (version 9.0.0; GraphPad Software, California, USA). For statistical comparison, data were expressed as mean ± standard deviation (SD). The BCVA was recorded as a logarithm of the minimum angle of resolution (LogMAR) equivalents. The Shapiro–Wilk test was used to assess the normality of counting variables before analysis. For quantitative variables with normal distribution, we performed one-way ANOVA and t-test; otherwise, the Kruskal–Wallis and Mann–Whitney U tests were used. Qualitative variables were analyzed with a chi-square test. Correlations between continuous variables were analyzed with Pearson’s correlation analyses; otherwise, Spearman’s correlation was used. Multiple linear regression analysis was used to determine the significant features associated with BCVA, in which case BCVA (LogMAR) was initially converted to normal distribution through a square root translation arithmetic. A *P*-value of < 0.05 was considered to be statistically significant.

## Results

A total of 137 subjects (231 eyes) were included in the study: 47 aCSC patients, 47 cCSC patients, and 43 healthy controls (shown in Table 1 and Fig. 1). There were 85 males and 52 females. Both sex and age were matched among the three groups (sex (male/female): 34/13 vs. 34/13 vs. 26/17, *P* = 0.381; age: 45.57 ± 7.28 vs. 48.43 ± 8.59 vs. 47.07 ± 5.06, *P* = 0.161). In the affected eyes, only 22 images of CC calculation were included, with 11 being aCSC and 11 being cCSC respectively.

**Table 1:** Basic characteristics among aCSC group, cCSC group, and healthy controls

	aCSC group, *n* = 47	cCSC group, *n* = 47	controls, *n* = 43	Statistical Value	*P* value
Male/Female	34/13	34/13	26/17	χ2 = 1.932	0.381^&^
Age (y)	45.57 ± 7.28	48.43 ± 8.59	47.07 ± 5.06	F = 1.854	0.161^&^
**Affected eyes**					
ELM(-)/( +)	44/3	32/15		χ2 = 9.895	0.002^#^
EZ(-)/( +)	8/39	5/42		χ2 = 0.803	0.370^#^
RPE(-)/( +)	2/45	2/45		Fisher exact test	1^#^
CNV	0	13		χ2 = 15.086	< 0.001^#^
CFT (μm)	439.21 ± 160.50	328.74 ± 124.18	207.51 ± 18.03	F = 42.332	< 0.001^&^
SFCT (μm)	383.74 ± 91.37	393.53 ± 104.82	217.65 ± 34.26	H = 61.722	< 0.001^&^
BCVA(LogMAR)	0.13 ± 0.17	0.30 ± 0.27	0	H = 29.173	< 0.001^&^
**Fellow eyes**					
ELM(-)/( +)	0/47	0/47		-	-
EZ (-)/( +)	44/3	43/4		Fisher exact test	1^#^
RPE(-)/( +)	35/12	26/21		χ2 = 3.782	0.052^#^
CNV	0	0		-	-
CFT (μm)	225.23 ± 56.67	221.23 ± 25.05		F = 2.701	0.071^&^
SFCT (μm)	337.15 ± 91.29	333.36 ± 97.78		F = 31.361	< 0.001^&^
BCVA(LogMAR)	0.01 ± 0.03	0.02 ± 0.03		H = 3.783	0.025^&^

*P*^&^ represent a comparison among affected eyes, fellow eyes, and healthy controls

*P*# represents a comparison between affected eyes and fellow eyes

## Assessment in the affected eyes of CSC patients

A comparison of baseline characteristics (Table 1) showed that the cCSC group had a higher rate of ELM( +) (*P* = 0.002) than the aCSC group, while there was no difference in the presence of ELM ( +) or RPE ( +) (both *P* < 0.05). Thirteen secondary CNVs were detected in the cCSC group, while none were found in the aCSC group. As expected, both aCSC and cCSC groups had significantly higher CFT, SFCT, and BCVA(LogMAR) than healthy controls (all *P* < 0.001).

**Fig. 2:**
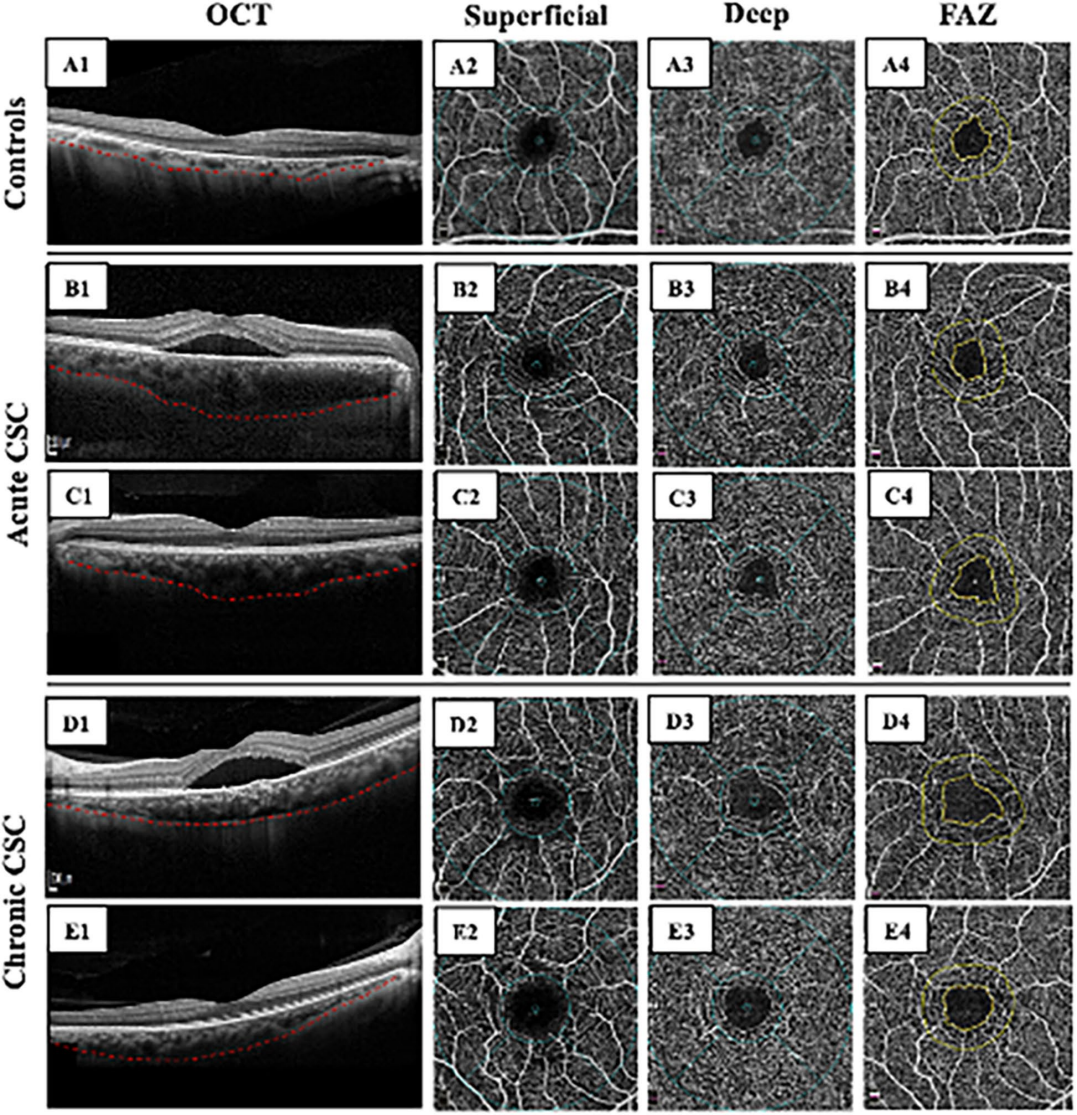
OCT and OCTA images of aCSC, cCSC, and healthy controls. A1-A4: healthy controls; B1-B4, C1-C4: the affected eye and the unaffected fellow eye of acute CSC patient, respectively; D1-D4, E1-E4: the affected eye and the unaffected fellow eye of chronic CSC patient, respectively.

The cCSC group had more decreased VD and FD-300 as well as a larger FAZ area (both the area and perimeter of FAZ) than healthy controls (all *P* < 0.05) (Shown in Table 2 and Fig. 2). However, only the aCSC group showed a lower SRVD (both *P* < 0.05) than healthy controls. There was no difference in AI among the three groups.

**Table 2:** OCTA parameters and comparison among affected eyes, fellow eyes, and healthy controls

			SRVD (%)							DRVD (%)					FAZ					
			Whole		Fovea		Parafovea			Whole		Fovea	Parafovea		Area(mm^2^)		Perimeter(mm)	AI	FD-300(%)	CC(%)
Affected eyes	aCSC		47.73±1.91		20.62±5.19		50.07±1.98			51.35±2.54		32.36±5.95	53.26±2.67		0.28±0.10		2.15±0.36	1.16±0.06	48.96±3.76	0.59±0.10
	cCSC		47.24±2.02		16.91±5.22		50.01±2.13			49.76±2.87		28.51±6.22	51.80±3.04		0.34±0.10		2.36±0.37	1.15±0.04	47.95±3.92	0.58±0.06
Fellow eyes	aCSC		48.31±2.11		17.90±5.89		51.15±2.35			50.90±2.76		31.49±6.24	52.98±2.69		0.30±0.10		2.15±0.37	1.13±0.03	50.11±3.38	0.63±0.06
	cCSC		47.79±1.84		16.02±4.44		50.74±1.95			50.10±2.54		29.50±5.30	52.40±2.63		0.33±0.08		2.31±0.29	1.14±0.05	50.67±2.75	0.64±0.05
Controls			48.79±1.49		20.03±5.28		51.64±1.58			51.19±3.11		32.46±4.66	53.28±3.20		0.29±0.07		2.16±0.24	1.14±0.04	51.04±3.25	0.64±0.06
*P* value among affected eyes of aCSC, cCSC, and healthy controls																				
*P *value		<0.001^***^		0.002^**^		<0.001^***^		0.014^*^			0.001^***^			0.026^*^		0.003^**^	0.003^**^	0.369	<0.001^***^	0.038^*^
aCSC VS cCSC		0.201		0.001^**^		0.889		0.008^**^			0.001^***^			0.019^*^		0.001^**^	0.015^*^	0.608	0.251	0.716
aCSC VS Controls		0.007^**^		0.594		<0.001^***^		0.798			0.933			0.978		0.674	0.996	0.608	0.068	0.93
cCSC VS Controls		<0.001^***^		0.005^**^		<0.001^***^		0.019^*^			0.001^***^			0.02^*^		0.007^**^	0.008^**^	0.347	<0.001^***^	0.037^*^
*P *value among fellow eyes of aCSC, cCSC, and healthy controls																				
*P* value		0.04^*^		0.002^**^		0.103			0.159		0.034^*^			0.33		0.044^*^	0.019^*^	0.565	0.647	0.752
aCSC VS cCSC		0.177		0.085		0.315			0.17		0.08			0.327		0.046^*^	0.064	0.418	0.666	0.625
aCSC VS Controls		0.217		0.056		0.246			0.616		0.401			0.617		0.717	0.999	0.309	0.349	0.458
cCSC VS Controls		0.011^*^		<0.001^***^		0.033^*^			0.066		0.011^*^			0.146		0.021^*^	0.022^*^	0.884	0.619	0.795

^*^P <0.05

^**^P <0.01

^***^P <0.001

Parameters of FD-300, AI, and CC were analyzed through the Kruskal-Wallis test, while other parameters were analyzed through a one-way ANOVA test

**Fig. 3:**
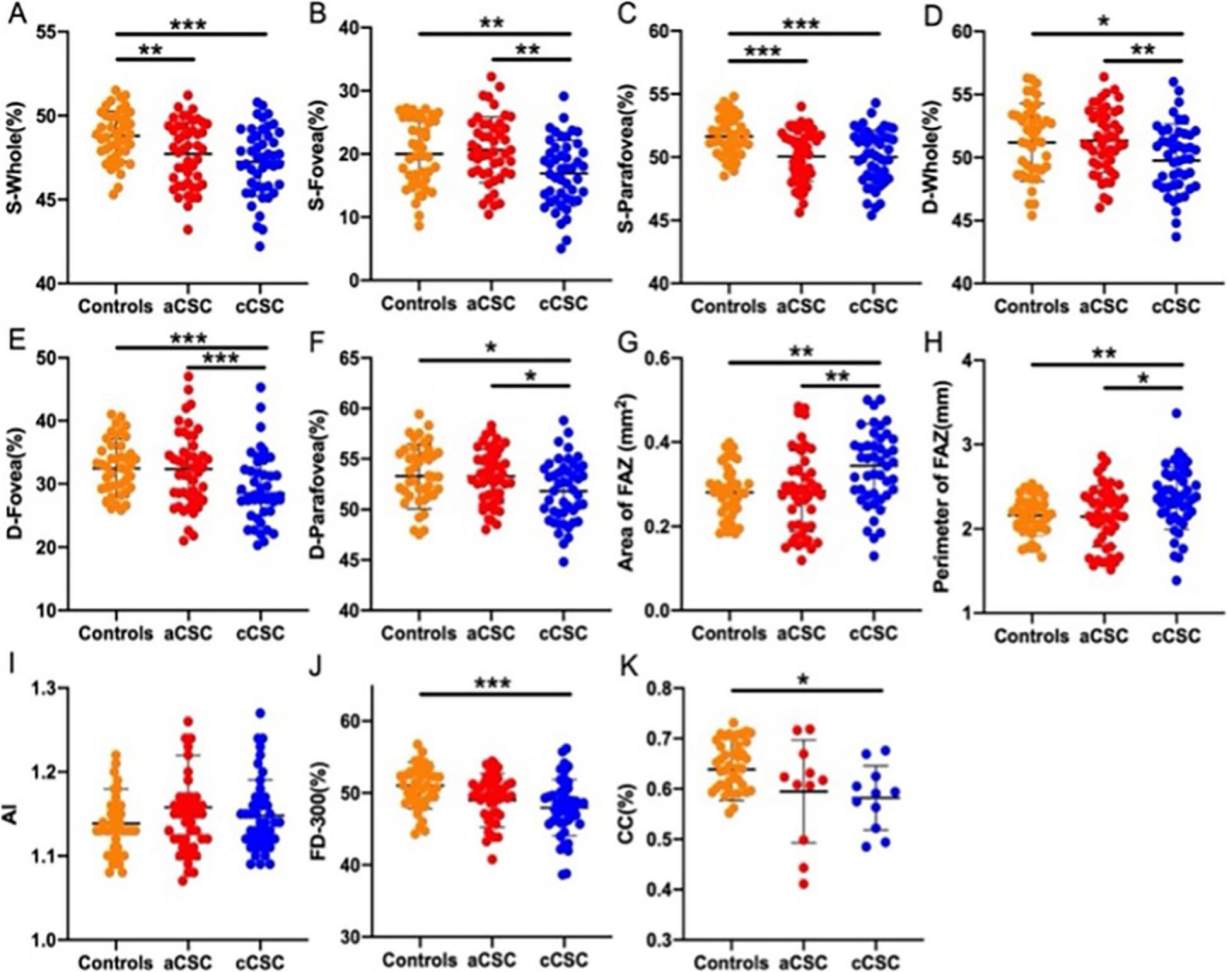
OCTA parameters of the affected eyes in aCSC and cCSC group, and healthy controls. **A**-**C **retinal VD of whole, fovea, and parafovea in the superficial layer, respectively; **D**-**F **retinal VD of whole, fovea, and parafovea in the deep layer, respectively; **G**-**J **FAZ parameters, including the area of FAZ, the perimeter of FAZ (PERIM), the A-circularity index (AI, perimeter/standard circle perimeter with equal area), the FD-300 (vessel density around the 300μm width of the FAZ), respectively; K: VD of CC layer. *P<0.05; **P<0.01; ***P<0.001

Spearman’s correlation analyses showed that patients with cCSC, EZ( +), and ELM( +) were more prone to have a lower VA (all *P* < 0.05; R = 0.396, R = 0.277, R = 0.331, respectively). Among the dependent and continuous parameters of OCT, OCTA, age, and BCVA, a statistical correlation with BCVA was not found in the aCSC group. In the cCSC group, DRVD-Fovea (*P* = 0.006) and FD-300 (*P* = 0.002) were strongly associated with BCVA, which could be expressed as a multiple linear regression equation: Y (BCVA) = 3.692–0.036✱X1 (DRVD-Fovea)-0.031✱X2 (FD-300), R2 = 0.427 (shown in Table 3 and Fig. 3). Pearson’s correlation analyses revealed that CFT was positively correlated with SRVD-Fovea and DRVD-Fovea (both *P* < 0.001; R = 0.402, R = 0.392, respectively), but negatively correlated with the area and perimeter of FAZ (both P < 0.05; R = -0.318, R = -0.282, respectively). There was no significant correlation between SFCT and any OCTA parameters.

**Table 3:** Multiple linear regression analysis of OCTA features that were strongly associated with BCVA in affected cCSC eyes

Features	t	*P* value
SRVD-Fovea	1.419	0.164
DRVD-Fovea	-2.89	0.006
DRVD-Parafovea	-3.287	0.09
FD-300	-1.737	0.002

## Assessment in the unaffected fellow eyes of CSC patients

Regarding basic characteristics (Table 1), ELM ( +) or secondary CNV was not found in either the aCSC or cCSC group. Moreover, 25.5% and 44.7% RPE ( +) were observed in the aCSC and cCSC groups (*P* = 0.052), respectively, which resulted in the rate of EZ ( +) of up to 6.4% and 8.5% (*P* = 0.694), respectively. Among the three groups, the aCSC group had higher CFT than healthy controls (*P* = 0.027), while there was no difference in CFT between the cCSC group and healthy controls (*P* = 0.087). Moreover, both aCSC and cCSC groups had significantly higher SFCT and BCVA(LogMAR) than healthy controls (*P* < 0.001, *P* = 0.25).

**Fig. 4:**
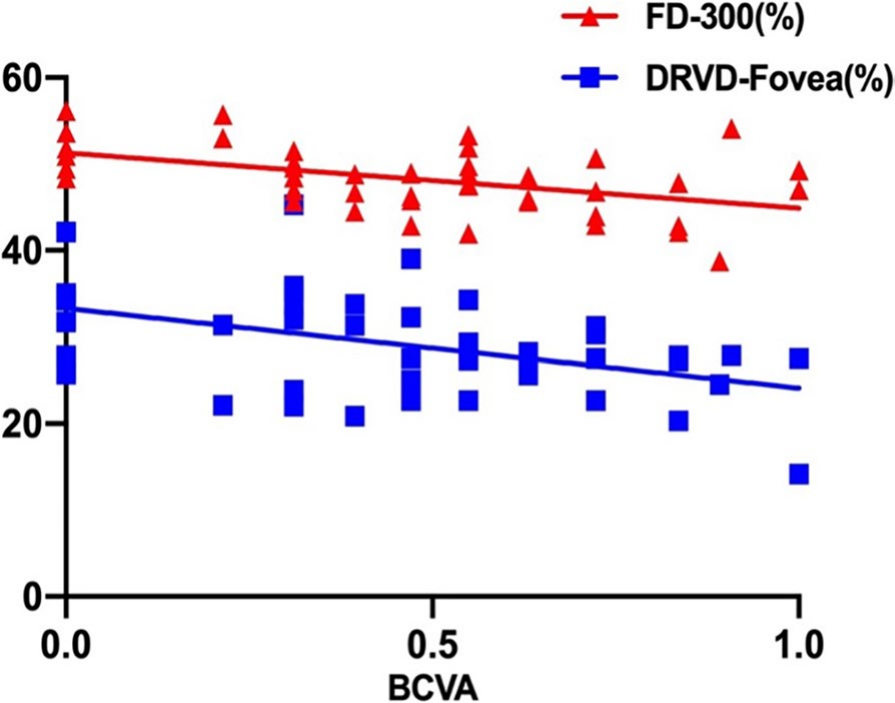
Multiple linear regression analysis: BCVA versus DRVD-Fovea and FD-300. BCVA=3.692-0.036✱(DRVD-Fovea)-0.031✱(FD-300), R^2^=0.427; BCVA(LogMAR) was converted to normal distribution through square root translation arithmetic during the analysis

As shown in Table 2 and Fig. 4, there was no significant difference between the aCSC group and healthy controls in all OCTA parameters. Surprisingly, all SRVD and DRVD-Fovea were significantly lower (all *P* < 0.05), and the two FAZ area parameters (area and perimeter) were significantly larger (all *P* < 0.05) in the cCSC group than in healthy controls, while there was no difference in the CC layer.

Among all variables presented in Table 2, the statistical correlation with BCVA was not found (all *P* > 0.05). However, Pearson’s correlation analyses revealed that CFT was negatively correlated with the VD of the CC layer (*P* = 0.017, R = -0.253). Significant correlation was not found between SFCT and any OCTA parameters.

## Assessment in both eyes of CSC patients

As reported in Table 4, the affected eyes of both aCSC and cCSC groups had a higher CFT, SFCT, and BCVA(LogMAR) than the fellow eyes (all *P* < 0.05). Regarding OCTA parameters, SRVD-Whole, SRVD-Parafovea, and FD-300 of the affected eyes showed a decline with higher severity than the fellow eyes in the aCSC group (all *P* < 0.05), while both the VD of the deep layer and the CC layer and FAZ showed no difference (all *P* > 0.05). In the cCSC group, SRVD-Parafovea and FD-300 were significantly lower in the affected eyes, while the VD of the CC layer was significantly higher (all *P* < 0.05).

**Table 4:** *P* value of OCTA parameters in aCSC and cCSC eyes after paired test

	SRVD (%)			DRVD (%)			FAZ								
*P* value	Whole	Fovea	Parafovea	Whole	Fovea	Parafovea	Area(mm^2^)	Perimeter(mm)	AI	FD-300(%)	CC(%)	CFT		SFCT	BCVA
aCSC	0.028^*^	<0.001^***^	<0.001^***^	0.22	0.058	0.432	0.12	0.874	0.01^*^	0.034^*^	0.139	<0.001^*^^*^^*^	0.001^**^		<0.001^***^
cCSC	0.084	0.107	0.027^*^	0.44	0.098	0.184	0.211	0.197	0.086	<0.001^***^	0.034^*^	<0.001^***^	<0.001^***^		<0.001^***^

^*^P<0.05

^**^P<0.01

^***^P<0.001

Parameters of FD-300, AI, CC, CFT, SFCT, and BCVA were analyzed through the Mann-Whitney U test, while other parameters were analyzed through paired t-test

No significant difference was observed between cCSC-CNV and none cCSC-CNV eyes in the baseline characteristics, OCTA, or OCT parameters.

## Discussion

Our study revealed some interesting findings: 1. Both the affected eyes and the unaffected fellow eyes of the cCSC group had the lowest retinal VD and the largest FAZ area among the three groups, which were significant when compared with healthy controls; 2. A lower VD of the CC layer was observed in the affected eyes of the cCSC group than in healthy controls; 3. There was no significant difference between the fellow eyes of the aCSC group and healthy controls in VD or FAZ, while the affected eyes showed a lower SRVD; 4. In the affected eyes, a multiple linear regression equation was found to speculate BCVA with DRVD-Fovea and FD-300, while no statistical correlation with BCVA was found in the fellow eyes. Given that both decreased VD and expanded FAZ were observed in cCSC patients in our previous study [10], such changes in the fellow eyes were still surprising. Other morphological observations such as RPE ( +) and EZ ( +) were noted in the fellow eyes of CSC patients, which further confirmed the injury.

Previous research has shown the pathological mechanism of retinal injury in CSC patients. Tomasso L [12] reported reduced retinal venous dilation in response to flicker-light stimulation in cCSC eyes, as a result of retinal venous stasis. Morphologically, Vogel RN [13] observed a type of cellular cluster resembling macrophages in the inner retina of eyes with active and resolved CSC by using adaptive optics scanning light ophthalmoscopy. Thus far, OCTA research involving comprehensive parameters (VD and FAZ) in CSC patients, especially in the fellow eyes, has been rare. Yu [14] found decreased superficial microvascular density and superficial total microvascular density in CSC patients (15 eyes), as did Cardillo PF (35 eyes) [15]. Karapapak M [16] detected a significant decline of VD in the symptomatic CSC eyes after the breath-holding maneuver, which indicated that the pathogenesis was related to an imbalance in local vascular regulation. Based on the current results that a significant difference between the fellow eyes of those with CSC and healthy controls, we also speculated that it was more likely a defective autoregulatory mechanism in both eyes of cCSC patients; the fellow eye might be at its preclinical status. However, how this trend changes during the follow-up observation and the relationship between further decreases in blood flow should be researched.

**Fig. 5:**
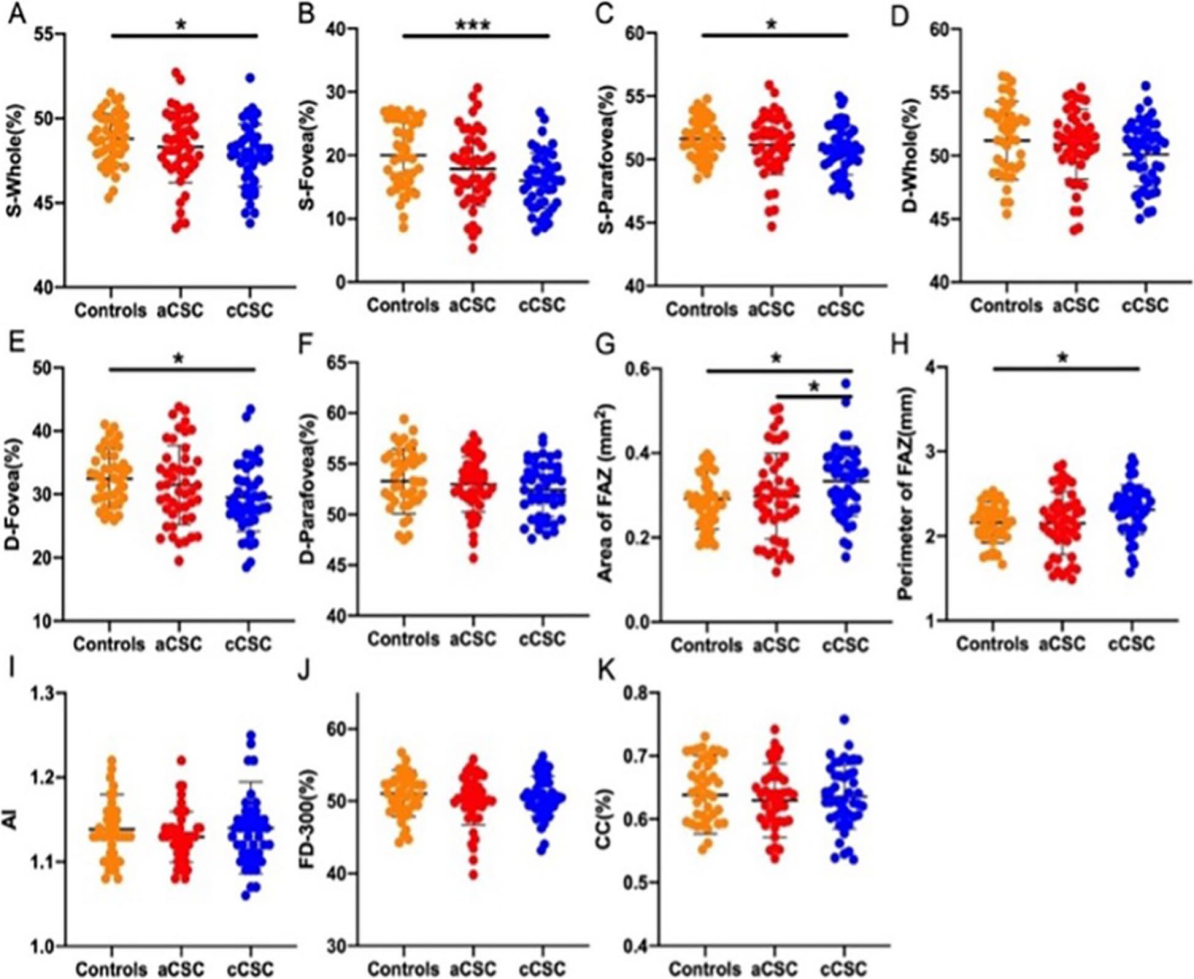
OCTA parameters of the unaffected fellow eyes in aCSC and CSC group, and healthy controls. **A**-**C **retinal VD of whole, fovea, and parafovea in the superficial layer, respectively; **D**-**F **retinal VD of whole, fovea, and parafovea in the deep layer, respectively; **G**-**J **FAZ parameters, including the area of FAZ, the perimeter of FAZ (PERIM), the A-circularity index (AI, perimeter/standard circle perimeter with equal area), the FD-300 (vessel density around the 300μm width of the FAZ), respectively; K: VD of CC layer. *P<0.05; **P<0.01; ***P<0.001

The hypoperfusion of the CC layer was also found, as observed in previous OCTA studies [6, 17]. In our study, considering that symptomatic SRDs in the affected eyes would be a major confounding factor to the measurement of the CC layer, we decided to calculate the VD of the remaining area as the real VD of the CC layer after the exclusion of any SRDs or RPE alterations in the image (Fig. 5). Despite the small sample of the affected eyes (11 eyes of aCSC and 11 eyes of cCSC), it could be the longer disease course that caused the significant hypoperfusion in cCSC, but not in the aCSC group or the fellow eyes. However, no correlation was found between the VD of the retina and the CC layer (all *P* > 0.05), and their exact pathology in CSC remains unknown.

Our results also showed that we could predicate BCVA with DRVD-Fovea and FD-300 in the affected eyes of cCSC patients. Anatomically, the SCP is located on the inner retina and primarily nourishes the inner layers, while the DCP resides adjacent to the outer nuclei layer, which is composed of highly oxygen-dependent photoreceptor axon terminals [18, 19]. On the other hand, the FAZ has the highest condensed cone photoreceptor density and oxygen consumption. It was clear that a decrease in VD around the Fovea part would impede signal transduction and cause damage to photoreceptor cells, which would lead to a worse VA. All CSC-affected eyes had an intact inner retinal structure, with no disruption or abnormal figuration of the FAZ observed. Since aCSC patients and healthy controls had no significant difference for most OCTA variables in this study (only SRVD changes in the affected eyes), we suggest that the configuration of the SRDs caused higher tension on the SCP than on DCP, leading to a greater abnormality.

To our knowledge, this is the first study to take VD of the retina and CC layer and the FAZ into account in the following eyes of those with CSC by providing comprehensive parameters both in OCTA and OCT. However, there are still some limitations. Considering that we used OCTA to measure retinal VD, we did not match the axis or diopter in all subjects [20]. Although our study aims to compare the fellow eyes of unilateral CSC at the initial examination with the affected eyes and healthy controls, the symptom duration time of our patients was based on their main complaint, which obscured the exact duration time. We also failed to observe the variations in the OCTA parameters within the follow-up time.

## Conclusions

In summary, by using OCTA and its self-software, we showed that both the fellow eyes and the affected eyes of cCSC patients had significantly lower VD of the retina and a larger FAZ area than healthy controls, which indicated that the asymptomatic fellow eyes may also suffer from the disease and should arouse our attention.

## Data Availability

The datasets used and analyzed during the current study are available from the corresponding author upon reasonable request.

## Abbreviations

VD: Vessel density
CC: Choriocapillaris
FAZ: Foveal avascular zone
CSC: Central serous chorioretinopathy
aCSC: Acute CSC
cCSC: Chronic CSC (cCSC)
OCTA: Optical coherence tomography angiography
OCT: Optical coherence tomography
DRVD: Deep retinal vessel density
SRVD: Superficial retinal vessel density
RPE: Retinal pigment epithelium
FFA: Fundus fluorescein angiogram
BCVA: Best corrected visual acuity
CNV: Choroidal neovascularization
SRDs: Serous retinal detachments
PEDs: Pigmented epithelium detachments
ICGA: Indocyanine green angiography
PDT: Photodynamic therapy
PERIM: The perimeter of FAZ
AI: The A-circularity index
FD-300: Vessel density around the 300 μm width of the FAZ
Anti-VEGF: Anti-vascular endothelial growth factor
CFT: Central foveal thickness
SFCT: Subfoveal choroidal thickness
ELM: External limiting membrane
EZ: Ellipsoid zone
SD: Standard deviation
BCVA: Logarithm of the minimum angle of resolution
